# Supplementary material for: Novel ATP2A2 Gene Mutation c.118G>A Causing Keratinocyte and Cardiomyocyte Disconnection in Darier Disease
Source: Biomedicines. 2024 May 10;12(5):1060. doi: 10.3390/biomedicines12051060 (PMC11118942; doi:10.3390/biomedicines12051060)
Supplement: Supplementary file 1 [file biomedicines-12-01060-s001.zip › biomedicines-2915860-supplementary.pdf]

Novel *ATP2A2* Gene Mutation c.118G>A Causing Keratinocyte and Cardiomyocyte Disconnection in Darier Disease.

SUPPORTING INFORMATION

Supplementary Figure S1. *List of the cardiac genes included in the next-generation sequencing clinical exome panel analyzed in this study*.

Supplementary Figure S2. *Integrative genomics viewer (Illumina) software visualization of the ATP2A2 gene c.118G>A variant.*

Supplementary Figure S3. *In silico* analysis of the *ATP2A2* c.118G>A variant using various splicing prediction programs.

Supplementary Figure S4: *Further ultrastructural details of enlarged spaces and absence of contacts between myocardiocytes in myocardium from Darier patient (B) as compared to the control (A).*

Supplementary Figure S5: *Western blot analysis of Serca-2*

Figure S1. List of the cardiac genes included in the next-generation sequencing clinical exome panel analyzed in this study.

ABCC9, ACTC1, ACTN2, AKAP9, ALMS1, ANK2, ANKRD1, BAG3, BRAF, CACNA1C, CACNA2D1, CACNB2, CALM1, CALM3, CASQ2, CAV3, CHRM2, CRYAB, CSRP3, CTNNA3, DES, DMD, DOLK, DSC2, DSG2, DSP, DTNA, EMD, EYA4, FHL1, FKRP, FKTN, FLNC, GAA, GATA4, GATA6, GATAD1, GJA5, GLA, GNB5, GPD1L, HCN4, HFE, HRAS, ILK, JPH2, JUP, KCND3, KCNE1, KCNE1L, KCNE2, KCNE3, KCNJ2, KCNJ5, KCNJ8, KCNQ1, KRAS, LAMA4, LAMP2, LDB3, LMNA, MAP2K1, MAP2K2, MURC, MYBPC3, MYH6, MYH7, MYL2, MYL3, MYLK2, MYOZ2, MYPN, NEBL, NEXN, NKX2-5, NRAS, PDLIM3, PKP2, PLN, PRKAG2, PTPN11, RAF1, RANGRF, RBM20, RYR2, SCN10A, SCN1B, SCN2B, SCN3B, SCN4B, SCN5A, SGCD, SHOC2, SNTA1, SOS1, TAZ, TBX20, TCAP, TGFB3, TMEM43, TMPO, TNNC1, TNNI3, TNNT2, TPM1, TRDN, TRPM4, TTN, TTR, TXNRD2, VCL.

Novel *ATP2A2* Gene Mutation c.118G>A Causing Keratinocyte and Cardiomyocyte Disconnection in Darier Disease.

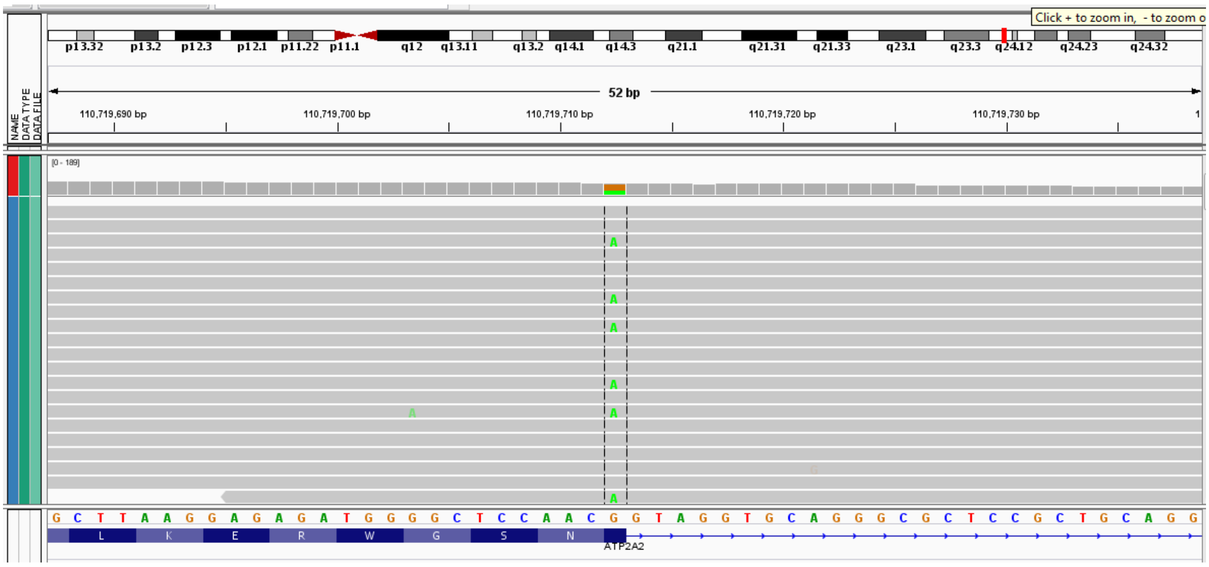

Figure S2. Integrative genomics viewer (Illumina) software visualization of the novel *ATP2A2* gene c.118G>A variant.

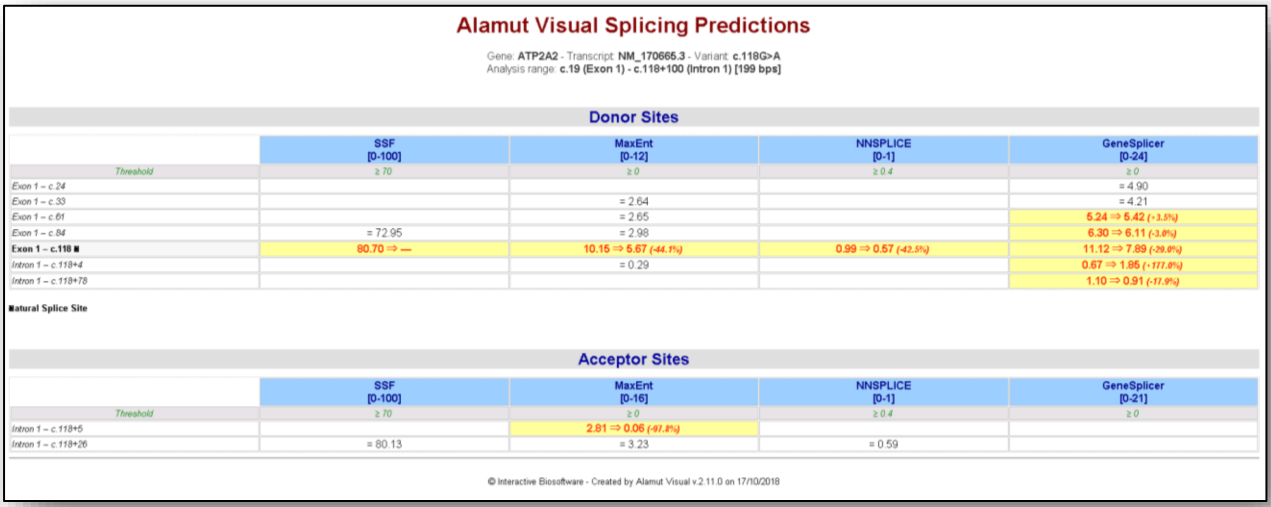

Alamut Visual Splicing Predictions

Gene: **ATP2A2** - Transcript: **NM_170665.3** - Variant: **c.118G>A**
Analysis range: **c.19 (Exon 1) - c.118+100 (Intron 1) [199 bps]**

Donor Sites

	SSF [0-100]	MaxEnt [0-12]	NNSPLICE [0-1]	GeneSplicer [0-24]
Threshold	≥ 70	≥ 0	≥ 0.4	≥ 0
Exon 1 – c.24				= 4.90
Exon 1 – c.33		= 2.64		= 4.21
Exon 1 – c.61		= 2.65		5.24 ⇒ 5.42 (+3.5%)
Exon 1 – c.84	= 72.95	= 2.98		6.30 ⇒ 6.11 (-3.0%)
**Exon 1 – c.118 ■**	80.70 ⇒ —	10.15 ⇒ 5.67 (-44.1%)	0.99 ⇒ 0.57 (-42.5%)	11.12 ⇒ 7.89 (-29.0%)
Intron 1 – c.118+4		= 0.29		0.67 ⇒ 1.85 (+177.0%)
Intron 1 – c.118+78				1.10 ⇒ 0.91 (-17.9%)

■ Natural Splice Site

Acceptor Sites

	SSF [0-100]	MaxEnt [0-16]	NNSPLICE [0-1]	GeneSplicer [0-21]
Threshold	≥ 70	≥ 0	≥ 0.4	≥ 0
Intron 1 – c.118+5		2.81 ⇒ 0.06 (-97.8%)		
Intron 1 – c.118+26	= 80.13	= 3.23	= 0.59	

© Interactive Biosoftware - Created by Alamut Visual v.2.11.0 on 17/10/2018

Figure S3. In silico analysis of the *ATP2A2* c.118G>A variant using various splicing prediction programs (Splice signal detection programs: SpliceSiteFinder-like [SSF], MaxEntScan [MaxEnt], NNSPLICE, and GeneSplicer) integrated in the Alamut software interface (Alamut version 2.10.0, Interactive Biosoftware, Rouen, France) showing the predicted consequences of c.954+1G>A variant at RNA level. *ATP2A2* c.118G>A mutation was predicted by all programs (SSF, MaxEnt, NNSPLICE, GeneSplicer) to severely decrease splicing efficiency of the donor site of exon 1 (SSF [–100.0%], MaxEnt [–44.1%], NNSPLICE [–42.5%], GeneSplicer [–29.0%]).

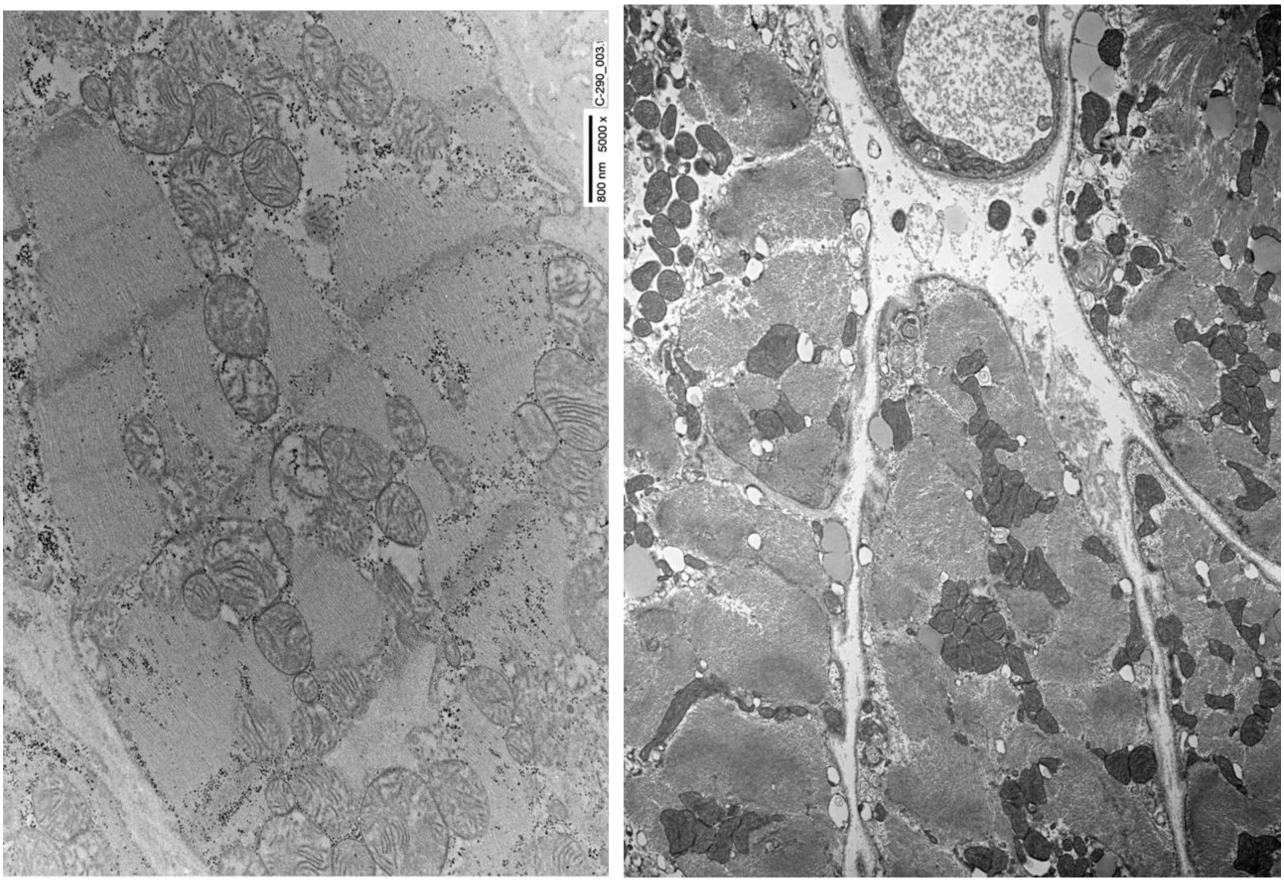

Figure S4. Further ultrastructural details of enlarged spaces and absence of contacts between myocardiocytes in myocardium from Darier patient (B) as compared to the control (A).

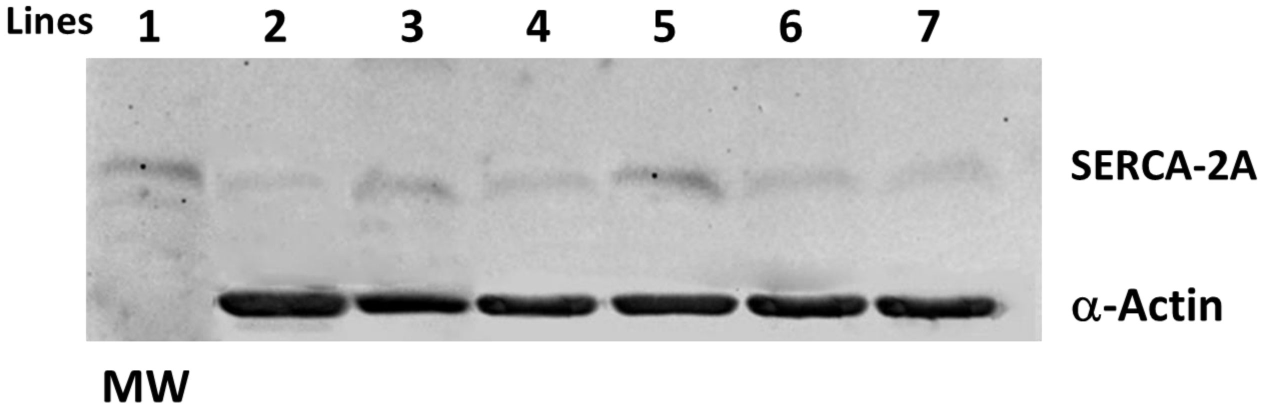

Figure S5. Upper: Western blot analysis of serca_2 (110 Kd) shows no significant quantitative differences between patient (line2) and surgical control biopsies.(line 3-6).p=0.106.
Lower: Alpha sarcomeric actin (43 kDa) was used as a loading control.
Line 1. Marker
